# Randomised controlled trial for evaluation of an ultrasound-guided palpation intervention for palpation skill training

**DOI:** 10.1038/s41598-022-05290-z

**Published:** 2022-01-24

**Authors:** Takashi Kitagawa, Yuma Aoki, Hotaka Sugimoto, Natsumi Ozaki

**Affiliations:** Department of Physical Therapy, School of Health Sciences, Shinshu University, 3-1-1 Asahi, Matsumoto, Nagano 390-8621 Japan

**Keywords:** Musculoskeletal system, Orthopaedics, Rehabilitation, Ultrasonography, Rehabilitation

## Abstract

Although there are increasing reports on the usefulness of sonopalpation with ultrasound imaging, many previous studies have reported interventions without a control group. This single-blind, parallel-group randomised controlled trial aimed to determine whether educational instruction with sonopalpation for physical therapy students has a more superior effect on skill improvement than traditional instruction without ultrasonography. Twenty-nine physical therapy students participated in the study and were randomised using block randomisation into an ultrasound imaging group (n = 15) and a control group (n = 14). Subsequently, they underwent three training sessions focusing on the shoulder joint. Participants underwent a scoring assessment of their palpation skills at pre-intervention, post-intervention, and follow-up 3 months after training. The raters were blinded to the subjects’ group. The Friedman and Mann–Whitney U tests were used for data analysis. The intervention group showed a significant increase in scores at post-intervention and the 3-month follow-up; the effect sizes were large (0.849 and 0.849, respectively). A comparison of the scores at different time points after the intervention at the 3-month follow-up revealed no significant difference between the groups. Education using ultrasound imaging may be non-inferior to education without it; nevertheless, further studies are needed to demonstrate superiority.

## Introduction

Obtaining accurate physical examination findings is one of the most basic and important skills for medical doctors and physical therapists. However, over the past 10 years, reports on palpation skills of medical doctors, physical therapists, and medical students have shown that the accuracy is generally low for each joint^1–3^. Accurate palpation of anatomical landmarks is imperative for an accurate diagnosis and/or establishment of the pathology or cause of pain^4^.

To address this problem, there have been increasing reports in recent years on the usefulness of ultrasound (US) imaging for palpation of musculoskeletal tissues^5,6^. Sonopalpation, a palpation technique that uses US imaging systems, is useful for diagnosing and identifying the cause of pain and improving the accuracy of diagnosis^7,8^. US imaging allows non-invasive and real-time evaluation compared with other diagnostic imaging techniques. It is used to identify anatomical landmarks in many departments, including the physical medicine and rehabilitation areas^9^. Additionally, systematic learning courses for improving palpation skills using US images have been developed to exploit US images recently^10^. Additionally, there has been an increasing number of interventional studies on the usefulness of US imaging as a tool to improve the palpation skills of medical students or residents^11,12^.

Among the many joints in the human body, the shoulder joint, in particular, is important for residents and physical therapists to palpate accurately to understand the cause of pain, dysfunction, and potential pathological causality^4^. It is estimated that approximately 20% of the population experiences shoulder pain^13^. For the management of such patients, proper identification and classification of the cause of pain are important. In addition, the ability to perform accurate palpation allows appropriate clinical assessment and interpretation of pathological conditions, such as injuries and abnormalities in the tissues surrounding the shoulder joint^14,15^. However, the insufficient palpation skills of medical students and inexperienced clinicians pose a problem.

Many preliminary studies have reported improvements in palpation skills using US imaging^5,6,10–12,16–18^. However, most of these were observational studies that did not examine confounders or interventional studies without a control group. Similarly, they had various limitations, such as insufficient blinding in an intervention trial. The results of these studies did not provide sufficient evidence for the efficacy of sonopalpation. In this study, we examined whether a palpation lecture using US images for physical therapy students is more useful in improving their skills than a traditional palpation lecture. We hypothesised that a course that uses US imaging findings would improve physical therapy students' shoulder joint palpation skills more than a course that focuses on the conventional method. We focused on the shoulder joint tissue because it is clinically important to accurately palpate this joint tissue; additionally, we sought to provide higher-quality evidence by conducting a single-blind randomised controlled trial.

## Methods

### Study design and participants

The study protocol conformed to the Declaration of Helsinki. The research was conducted with approval from the Medical Research Ethics Committee at Shinshu University (no. 4372), and our research protocol was registered with UMIN-CTR (trial number: UMIN000038286) on 15 October 2019.

Twenty-nine first-year college students from a single university, gathered through publicity via campus bulletin boards and other means, participated in a hands-on training course on palpation from 2019 to 2020. After the subjects were fully informed about the details regarding participation in this study, their written informed consent was obtained. The exclusion criteria for participants were as follows: (1) those with sensory impairment in the fingers; (2) physical therapy students who had worked in the medical or health-related field previously or who had previously taken a course to acquire palpation skills; and (3) any other person who was considered inappropriate as a subject by the principal investigator for valid reasons. Data collection and implementation were performed in the practical training room of the university. Of the 38 participants who received explanations, 29 who consented to participate in the study were randomly classified into two groups as follows: the US imaging-based sonopalpation education group (US group) and the conventional palpation education group (control group). Randomisation was performed using a block randomisation method with a block size of 4. The order of allocation was concealed until patients were fully allocated to each group. The order of assignment was made by those who were not intervention implementers or evaluators.

### Measurements

For each group of participants, before the course started, a scoring assessment of the participants' palpation skills in terms of accuracy and time was conducted with reference to previous studies^18^. In summary, the scoring test comprised an assessment of the accuracy and time required to palpate five tissues randomly selected for palpation by each participant from 30 tissues around the shoulder joint. To eliminate any selection bias, we used simple randomisation with a forced balance procedure to choose palpation targets a priori. Palpation accuracy was scored as palpation of the correct position of the target tissue without error, with 10 points for a perfect palpation, 5 points for an erroneous palpation at a distance of less than 1 cm, and 0 points for an erroneous palpation at a distance of more than 1 cm. Palpation speed was scored as the time required to palpate the target tissue. Ten points were assigned if the tissue was palpated in less than 10 s, 5 points if it was palpated between 10 and 30 s, and 0 points if it was palpated in more than 30 s. This scoring was performed for each of the five tissues, with a maximum of 50 points for accuracy and speed, for a total score of 100 points. The following 30 tissues were selected for instruction: scapula (scapular spine, acromion, scapular spine edge, medial border, superior angle, inferior angle, lateral border, infraglenoid tubercle of the scapula, and coracoid process), clavicle (shaft, acromioclavicular joint, and sternoclavicular joint), humerus (greater tubercle, lesser tubercle, and intertubercular groove), and muscles around the shoulder joint (deltoid, pectoralis major, supraspinatus, infraspinatus, teres minor, teres major, subscapularis, latissimus dorsi, coracobrachialis, trapezius, rhomboid, levator scapulae, pectoralis minor, serratus anterior, and biceps brachii). Students were informed before and after their testing to avoid sharing the details of the assessment with other students. The primary outcomes of the study were the accuracy score, speed score, and combined total score.

After completing the initial assessment, participants attended a course on palpation of the shoulder joint (90 min × 3 sessions in total) at 1, 3, and 5 weeks. To standardise the interventions, the target tissues for instruction in the three lectures were chosen as the 30 tissues, as described above. For the US group, the palpation training was conducted by providing visual feedback using US images taken by a 5–18-MHz linear probe. Two US imaging systems (ARIETTA Prologue, Hitachi Aloka Medical, Tokyo, Japan and Venue40 MSK, GE Healthcare, Tokyo, Japan) were utilised. The participation schedule was adjusted such that five participants attended each course^9^. In principle, no tailoring of the course was performed for any participant. Because of the nature of the intervention, we could not blind the participants and the administrator. However, to reduce bias, the outcome evaluators were not informed of the participants’ assigned group.

**Figure 1:**
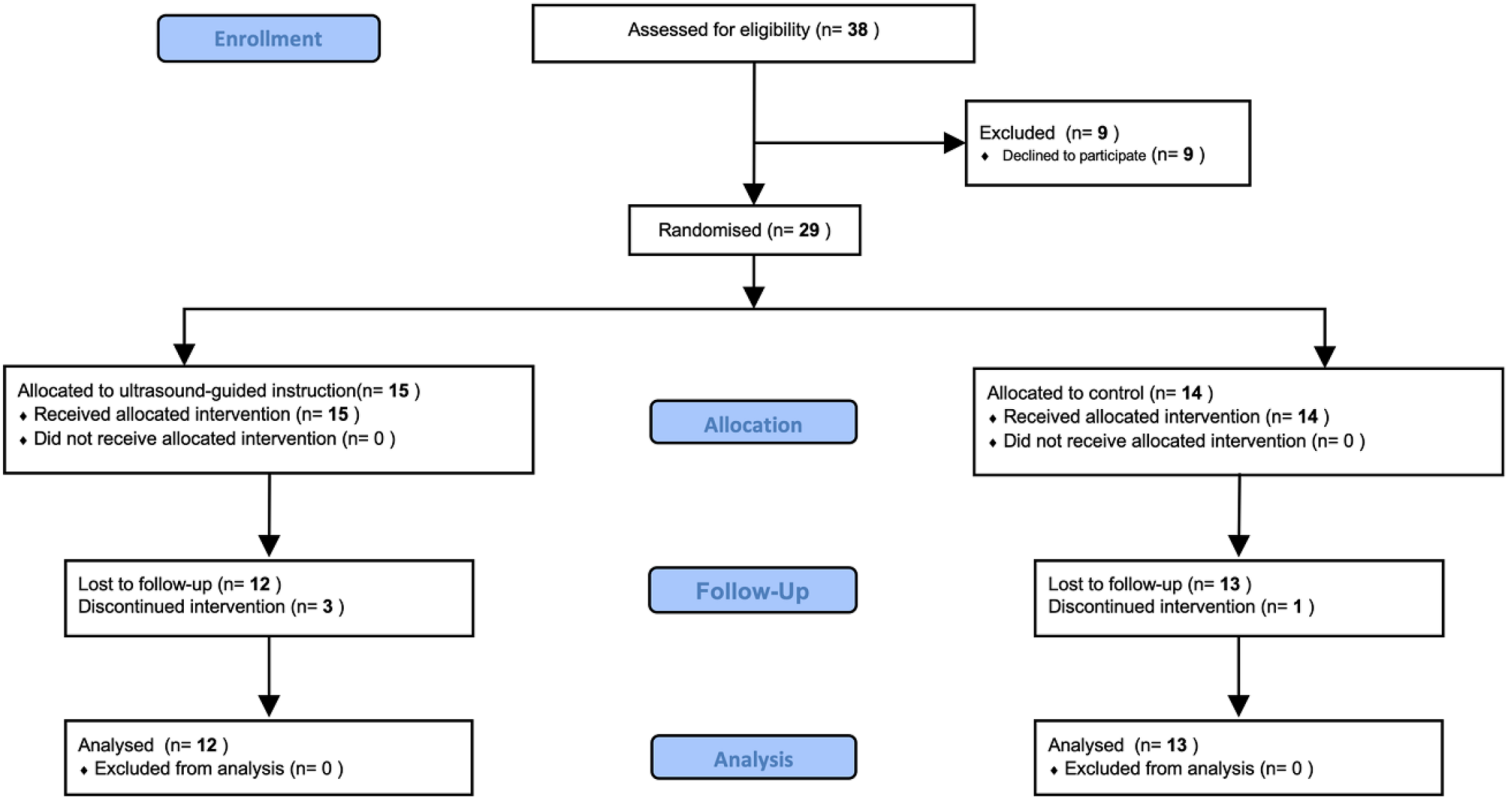
CONSORT flow diagram of the study.

One week after the end of the intervention, the students participated again in a scoring assessment of their palpation skills. Further assessment was conducted 12 weeks after the end of the course to confirm the lasting effects (Fig. 1). Similarly, the scores were compared between the three assessment time points: (1) pre-intervention (T1)—immediately post-intervention (T2), (2) immediately post-intervention (T2)—12-week follow-up (T3), and (3) pre-intervention (T1)—12-week follow-up (T3). During the period from before the start of the intervention to the follow-up assessment, the participants were instructed to not perform any special practical exercises to ensure consistency.

### Statistical analysis

To examine the changes within each group and at the time points T1–T2, T2–T3, and T1–T3, comparisons between the corresponding groups were conducted using the Friedman test. If significant differences were found, the multiple comparison method (Bonferroni method) was used to examine the changes according to the predetermined time points. At T2 and T3, the Mann–Whitney U test was used to compare the scores between the groups. To examine the amount of change with each intervention and time point, Cohen d effect sizes were calculated for each test that showed a significant difference^19^. To manage missing data, simple imputation (mean imputation) was used for both missing data at random and otherwise. The sample size was calculated using G*Power 3.1.9.7 (Heinrich Heine University, Düsseldorf, Germany). For the non-paired comparison between groups of non-normally distributed indices, each group was calculated to require 51 participants, assuming an effect size of 0.5, a power of 0.8, and a significance level of less than 5%. Due to the impact of coronavirus disease 2019 (COVID-19), we could not recruit the required number of participants for the sample size. As the pandemic was expected to be prolonged, it became difficult to gather the number of participants initially planned within a short period even if the recruitment period was extended; thus, the experiment was terminated with the number of participants initially recruited, and the analysis was conducted. In the post-hoc analysis, the power was 0.33, and SPSS Statistics 27.0 (IBM Corp., Armonk, NY, USA) was used to perform the statistical analyses.

## Results

### Participant flow

The recruitment period was 2019–2020, and the follow-up period was up to 3 months following the first intervention (March 2021). Of the 38 first-year physical therapy students, 29 (76.3%) participated in the study. It seemed difficult to continue the recruitment of new participants due to the lack of certainty regarding the COVID-19 pandemic. Therefore, this study was suspended in March 2021. The participants included 11 men and 18 women (median age: 20 years old). The baseline scores for each group were similar (Table 1). The 29 participants were randomly assigned to the two groups, and three participants in the US group and one participant in the control group dropped out during the follow-up period (Fig. 1). Because the study was conducted over 2 years, 2019 and 2020, there was a 1-year time gap between the start of the intervention for approximately half of the study participants. During that time, there were no major changes in other classes or educational curricula. During the intervention, the number of students attending one lecture was limited because of the restriction that up to five students per teacher were eligible for lectures using US images. Therefore, there was a lag of less than 3 h between randomisation and the start of the intervention, depending on the group assigned. The actual intervention was performed as planned, with a fixed and formulated flow with a short time lag. Finally, 25 participants who were followed up (12 in the US group and 13 in the control group) were included in the analysis.

**Table 1:** Palpation outcome scores for participants at baseline.

Characteristics	US group (n = 15)	Control group (n = 14)
Accuracy score at baseline	20 (15–25)	27.5 (15–35)
Speed score at baseline	20 (10–25)	22.5 (10–25)
Total score at baseline	40 (30–47.5)	50 (20–60)

### Comparison of each score

**Figure 2:**
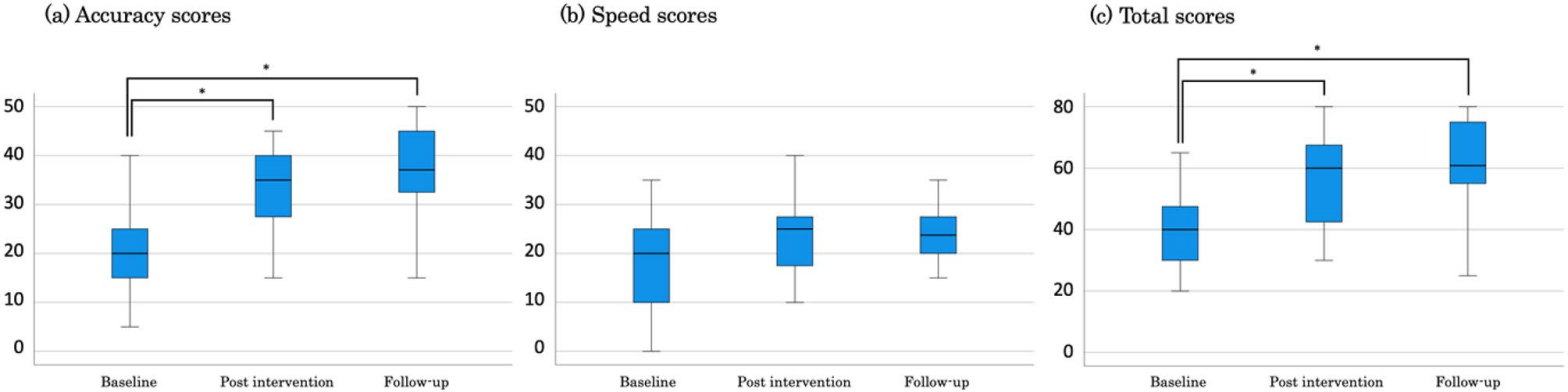
Palpation scores at each time point for the ultrasound group. *: p < 0.05.

**Figure 3:**
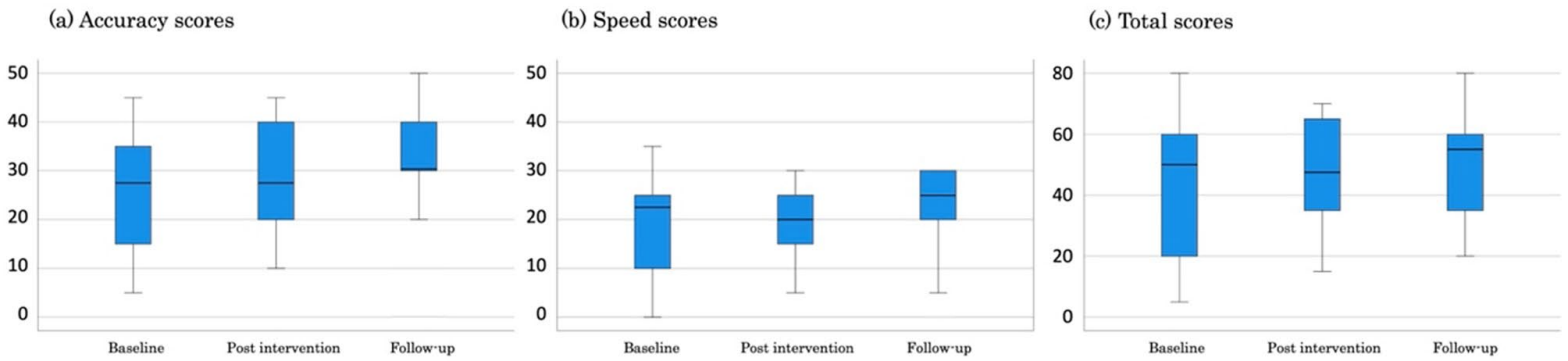
Palpation scores at each time point for the control group.

Analyses of the assessment scores showed multiple significant differences at each time point in the US group (Fig. 2a–c). However, no significant difference was observed in the control group at any of the time points (Fig. 3a–c). In the US group at T1, the median accuracy score was 20.0 (range, 15–25), the median speed score was 20 (range, 10–25), and the median total score was 40 (range, 30–47.5) (Fig. 2a). In the US group at T2, the median accuracy score was 35.0 (range, 27.5–40), the median speed score was 25.0 (range, 17.5–27.5), and the median total score was 60.0 (range, 32.5–45) (Fig. 2b). In the US group at T3, the median accuracy score was 37.1 (range, 32.5–45), the median speed score was 23.8 (range, 20–27.5), and the median total score was 60.8 (range, 55–75) (Fig. 2c). In the control group at T1, the median accuracy score was 27.5 (range, 15–35), the median speed score was 22.5 (range, 10–25), and the median total score was 50 (range, 20–60) (Fig. 3a). In the control group at T2, the median accuracy score was 27.5 (range, 20–40), the median speed score was 20 (range, 15–25), and the median total score was 47.5 (range, 35–65) (Fig. 3b). In the control group at T3, the median accuracy score was 30.4 (range, 30–40), the median speed score was 25 (range, 20–30), and the median total score was 55 (range, 35–60) (Fig. 3c). The effect sizes for the time points for which significant differences in the accuracy score were noted were 0.849 for T1–T2 and 0.990 for T1–T3 in the US group. The effect sizes for the total score were 0.849 for T1–T2 and 0.849 for T1–T3 in the US group. Comparison of scores between the groups at T2 and T3 showed no significant differences at either time point (Table 2). No adverse events were observed in either group.

**Table 2:** Comparison of each palpation score at post-intervention and follow-up.

Characteristics	US group (n = 12)	Control group (n = 13)	*P* value
**Palpation scores at T2 (post-intervention)**			
Accuracy score	35 (27.5–40)	27.5 (20–40)	0.234
Speed score	25 (17.5–27.5)	20 (15–25)	0.234
Total score	60 (42.5–67.5)	47.5 (35–65)	0.186
**Palpation scores at T3 (follow-up)**			
Accuracy score	37.1 (32.5–45)	30.4 (30–40)	0.158
Speed score	23.8 (20–27.5)	25 (20–30)	0.847
Total score	60.8 (55–75)	55 (35–60)	0.102

## Discussion

Our results demonstrated that the US group showed better improvement in skills due to the intervention, while the control group did not show any meaningful improvement. Another finding of this study was that the impact of the intervention in the US group was relevant in the short and medium terms. However, the comparisons of palpation scores between the two groups showed no significant differences at each time point.

Many previous studies supported the use of US imaging in improving palpation skills. Improved palpation skills have been reported using US imaging at the lumbar spine^11^, shoulder joint^5^, some body landmarks^12^, various upper extremity landmarks^10^, and knee joint landmarks^6^. However, these were only intervention studies without control groups, and the differences between the new method and the usual instructional method could not be assessed.

Several randomised controlled trials have been conducted to overcome the limitations of these studies. Additionally, the results of these studies support the usefulness of ultrasonography, which has been reported to be useful for the shoulder and knee joints^16^, elbow joint, and ankle joints^18^. However, most previous studies examined the immediate effects after the intervention and did not examine the lasting effects. In the present study, to overcome the limitations of previous studies, we conducted a randomised controlled trial with medium-term follow-up from immediately post-intervention to 3 months. To our knowledge, this is the first study to examine the medium-term effects of the utilisation of US imaging for improving palpation skills. The effect size also indicated a moderate effect.

The results of the scoring data analysis showed a significant improvement in the accuracy of palpation, rather than speed, leading to an increase in the overall score. Using US images, students can learn the palpation technique by obtaining visual feedback^5,17^. This might be one of the reasons why the students recruited in our study obtained more accurate palpation skills for each landmark. Palpation using US images is also called ‘sonopalpation’^7,8^. Accurate palpation of the anatomical location and accurate examination of the tenderness of soft tissue are very important clinically to identify the tissue causing pain. The importance of palpation skills using US images is expected to increase in the future, and it is desirable to construct a systematic curriculum that includes US imaging education of whole-body landmarks^10^. In contrast, it has been reported that the accuracy of palpation of the foot was still low even after training to improve palpation skills using ultrasonography^20^. This study mainly targeted the shoulder joint, which has been frequently examined in previous studies on palpation skills using US images. However, it is unclear whether the study results can be directly applied to other joints.

There was no significant difference between the groups at T2 and T3 (Table 2). Since there was no noticeable difference in the baseline scores, there may not be a relevant difference between these two intervention methods. Considering these advantages, purchasing a new device seems unnecessary. Additionally, it is not recommended that a US device being used for a certain purpose, such as abdominal diagnosis and medical check-up, be moved too far to another department with no US diagnostic equipment.

This study has some limitations. First, the evaluator's judgement may not have been perfectly accurate. However, a well-trained physiotherapist specialising in sports and musculoskeletal rehabilitation judged the accuracy of the palpation performed by the examinee students. Further, the physical therapists who evaluated the accuracy of palpation in this study regularly delivered lectures on palpation to young clinicians and students at universities and used US imaging. Nevertheless, in future studies, it would be better to use ultrasonography to confirm the training accuracy^4^. Second, since only one joint was used in this study, the external validity may have been reduced. A previous study reported low inter-rater reliability when comparing the agreement between 32 palpations of the long head biceps tendon using ultrasonography by two physical therapists with a certain level of experience^4^. In future studies, palpations of different body landmarks should be assessed. Third, participation in this study was voluntary; thus, there was a possibility of self-selection bias. Further, a perfect participation rate could not be expected. Additionally, the fact that this trial was conducted during a global pandemic may have contributed to the low participation rate. We should have taken all possible safety measures, such as sufficient infection control measures, and made announcements so that potential study recruits would have a strong sense of safety. Nevertheless, further studies that address the limitations of the current study are needed to clarify these points.

In conclusion, when compared with usual training courses, US-based training may improve the skills of physical therapy students, especially in terms of the accuracy of palpation. There were no invasive or adverse events associated with the use of US imaging equipment. On the contrary, since no clear differences between groups were observed in this study, we do not recommend that all educational programmes introduce US imaging devices but that the usefulness of ultrasonography should be evaluated based on the situation.

## Data Availability

The datasets generated during and/or analysed during the current study are available from the corresponding author on reasonable request.
